# Mosquitoes (Diptera: Culicidae) and their relevance as disease vectors in the city of Vienna, Austria

**DOI:** 10.1007/s00436-014-4237-6

**Published:** 2014-12-03

**Authors:** Karin Lebl, Carina Zittra, Katja Silbermayr, Adelheid Obwaller, Dominik Berer, Katharina Brugger, Melanie Walter, Beate Pinior, Hans-Peter Fuehrer, Franz Rubel

**Affiliations:** 1 Institute for Veterinary Public Health, Department for Farm Animals and Veterinary Public Health, University of Veterinary Medicine Vienna, Veterinärplatz 1, 1210 Vienna, Austria; 2 Institute of Parasitology, Department of Pathobiology, University of Veterinary Medicine Vienna, Veterinärplatz 1, 1210 Vienna, Austria; 3 Institute of Specific Prophylaxis and Tropical Medicine, Center for Pathophysiology, Infectiology and Immunology, Medical University of Vienna, Kinderspitalgasse 15, 1090 Vienna, Austria

**Keywords:** Culicidae, Species composition, Mosquito-borne disease, Urban, Vienna, Austria

## Abstract

Mosquitoes (Diptera: Culicidae) are important vectors for a wide range of pathogenic organisms. As large parts of the human population in developed countries live in cities, the occurrence of vector-borne diseases in urban areas is of particular interest for epidemiologists and public health authorities. In this study, we investigated the mosquito occurrence in the city of Vienna, Austria, in order to estimate the risk of transmission of mosquito-borne diseases. Mosquitoes were captured using different sampling techniques at 17 sites in the city of Vienna. Species belonging to the *Culex pipiens* complex (78.8 %) were most abundant, followed by *Coquillettidia richiardii* (10.2 %), *Anopheles plumbeus* (5.4 %), *Aedes vexans* (3.8 %), and *Ochlerotatus sticticus* (0.7 %). Individuals of the *Cx. pipiens* complex were found at 80.2 % of the trap sites, while 58.8 % of the trap sites were positive for *Cq. richiardii* and *Ae. vexans. Oc. sticticus* was captured at 35.3 % of the sites, and *An. plumbeus* only at 23.5 % of the trap sites. *Cx. pipiens* complex is known to be a potent vector and pathogens like West Nile virus (WNV), Usutu virus (USUV), Tahyna virus (TAHV), Sindbis virus (SINV), *Plasmodium* sp., and *Dirofilaria repens* can be transmitted by this species. *Cq. richiardii* is a known vector species for Batai virus (BATV), SINV, TAHV, and WNV, while *Ae. vexans* can transmit TAHV, USUV, WNV, and *Dirofilaria repens. An. plumbeus* and *Oc. sticticus* seem to play only a minor role in the transmission of vector-borne diseases in Vienna. WNV, which is already wide-spread in Europe, is likely to be the highest threat in Vienna as it can be transmitted by several of the most common species, has already been shown to pose a higher risk in cities, and has the possibility to cause severe illness.

## Introduction

Culicidae are of medical importance especially regarding mosquitos of the genera *Anopheles*, *Aedes*, and *Culex*—but also of other genera—which are known to transmit viruses, protozoa, and nematodes (Hubálek 2008; Sinka et al. 2010; Ledesma and Harrington 2011). Due to the (re-)emergence of several mosquito-borne diseases, like West Nile virus (WNV) or Usutu virus (USUV), those diseases and their vectors have become the main focus not only for many researchers (e.g., Gratz 1999; Takken et al. 2007; Hubálek 2008), but also for public health authorities (e.g., Vazquez-Prokopec et al. 2010).

From an anthropocentric point of view, the spread of diseases in urban areas is of special interest, as in developed countries, a large part of the human population lives in cities. In comparison to the surrounding rural regions, urban areas are characterized by habitat loss resulting in a reduced diversity of species (McKinney 2002). On the other hand, urban heat islands increase survival, breeding success, and activity of arthropod vectors (Bradley and Altizer 2007). Previous studies conducted in the USA already revealed the effect of urban land use on the abundance of Culicidae species. For example, the occurrence of *Culex pipiens*, the main WNV vector, is positively correlated with human population density and can be found in high abundances in urban areas (Pecoraro et al. 2007; Trawinski and MacKay 2010). This tendency is supported by the observation that urban trap sites contained more WNV positive pools than those from suburban sites (Deichmeister and Telang 2011).

The collection of data on the presence, absence, and abundance of vectors is an important factor to estimate the risk for the transmission of vector-borne diseases (Braks et al. 2011). However, data on the abundance of Culicidae are rare for European cities (Merdić et al. 2010; Krüger et al. 2014), and do not exist for Austria. The aim of this study is twofold: First, to investigate the species composition of Culicidae in the city of Vienna, Austria. Subsequently, a literature study was conducted to determine the importance of the captured species in the transmission of mosquito-borne diseases known for Austria, and discuss the possible risk for the spread of those diseases within the city of Vienna.

## Material and methods

### Study site

Vienna is the capital city of Austria and among the largest cities of Europe, with a population of about 1.8 million. Using the Corine Land Cover classes (CLC, level 2; EEA 2007) to describe this city, it comprised mostly of “urban fabric” (46.8 %), “forests” (18.0 %), “arable land” (9.8 %), “industrial, commercial, and transport units” (8.8 %), and “artificial, non-agricultural vegetated areas” (6.4 %). The climate is temperate, fully humid with warm summers, corresponding to Cfb climate following the Köppen-Geiger climate classification (Kottek et al. 2006), with 15 to 20 hot days per year (maximum temperature ≥30 °C). Thus, requirements for possible major outbreaks of vector-borne diseases are fulfilled for Vienna, especially under climate warming scenarios with increasing numbers of hot days.

### Mosquito trapping and identification

**Fig. 1:**
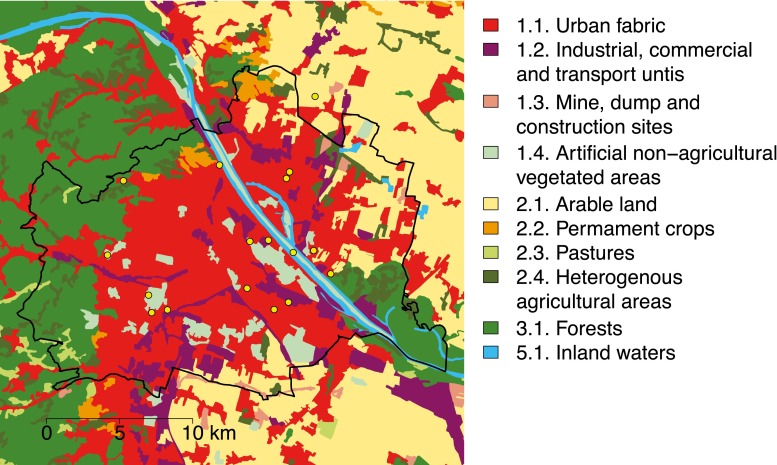
Location of the 17 trap sites (*yellow circles*) in the urban area of Vienna (*black line*) in relation to the Corine Land Cover classes (CLC level 2)

The present study combines the results of three Culicidae monitoring programs (2012 to 2014). The following sampling techniques to capture adult mosquitoes were used, namely, the new standard miniature light traps (John W. Hock Company, Gainesville, Florida) with an additional CO_2_ release, BG Sentinel traps (Biogents AG, Regensburg, Germany) equipped with CO_2_, and a specific lure (Sweet Scent^TM^), and exhausters. Samples were collected at 17 sites in Vienna (Fig. 1), with 1 to 489 trapping events per site (total number of trapping events = 1377). A trapping event lasted for 24 h, with the exception of samples done with the exhausters.

To account for site-specific differences in the trapping effort, which could bias the resulting proportions of captured species, the average number of each species per site and trapping event were calculated. These average values were further used to describe the average species composition of Vienna.

Female mosquitoes were identified using morphological characteristics according to the identification keys from Becker et al. (2010) and Schaffner et al. (2001). For this study, the different species were identified, with exception of mosquitoes from the *Culex pipiens* complex, the *Anopheles maculipennis* complex, and the species *Aedes cinereus*/*Aedes geminus*, as they cannot be reliably distinguished based on morphological characteristics only. For instance, in Europe, the *Cx. pipiens* complex is known to comprise of *Cx. pipiens pipiens* Linneaus, *Cx. pipiens* biotype *molestus* Forskal and *Culex torrentium* Martini (Becker et al. 2012).

## Results

Overall, we captured at the 17 sites 25,908 female Culicidae; in 24,736 cases, the species/complex could be identified (95.5 %). The captured females matched to 22 species of the genera *Anopheles*, *Aedes*, *Ochlerotatus*, *Culex*, *Culiseta*, *Coquillettidia*, and *Uranotaenia* (Table 1). On average, species belonging to the *Cx. pipiens* complex (78.8 %) were most abundant, followed by *Coquillettidia richiardii* (10.2 %), *Anopheles plumbeus* (5.4 %), *Aedes vexans* (3.8 %), and *Ochlerotatus sticticus* (0.7 %). Of those frequent species, the *Cx. pipiens* complex was present at 15 of the 17 trap sites, *Cq. richiardii*, and *Ae. vexans* were found at 10 sites. Most of the species were captured only at a few sites, while one third of the species only occurred at one specific trap site.

**Table 1:** Captured and identified Culicidae at the 17 trap sites in Vienna in the years 2012–2014

	Total		Average per site and trapping event		Species positive sites	
	Number	Percent	Number	Percent	Number	Percent
*Aedes cinereus*/*geminus*	14	0.1	0.00	0.0	3	17.6
*Ae. vexans*	4446	18.0	1.31	3.8	10	58.8
*Anopheles algeriensis*	9	0.0	0.00	0.0	1	5.9
*An. hyrcanus*	305	1.2	0.05	0.1	1	5.9
*An. maculipennis* complex	27	0.1	0.08	0.2	5	29.4
*An. plumbeus*	52	0.2	1.83	5.4	4	23.5
*Coquillettidia richiardii*	10,682	43.2	3.47	10.2	10	58.8
*Culiseta annulata*	15	0.1	0.03	0.1	4	23.5
*Culex martinii*	5	0.0	0.01	0.0	3	17.6
*Cx. modestus*	54	0.2	0.01	0.0	3	17.6
*Cx. pipiens* complex	8091	32.7	26.86	78.8	15	88.2
*Cx. territans*	6	0.0	0.01	0.0	4	23.5
*Ochlerotatus cantans*	1	0.0	0.00	0.0	1	5.9
*Oc. caspius*	27	0.1	0.00	0.0	3	17.6
*Oc. communis*	10	0.0	0.05	0.1	1	5.9
*Oc. dianteus*	3	0.0	0.01	0.0	3	17.6
*Oc. dorsalis*	2	0.0	0.12	0.3	1	5.9
*Oc. flavescens*	1	0.0	0.00	0.0	1	5.9
*Oc. geniculatus*	35	0.1	0.01	0.0	4	23.5
*Oc. intrudens*	10	0.0	0.01	0.0	3	17.6
*Oc. sticticus*	935	3.8	0.25	0.7	6	35.3
*Uranotaenia unguiculata*	6	0.0	0.00	0.0	1	5.9
Total	24,736	100.0	34.10	100.0	17	100.0

## Discussion

In the present study, mosquitoes of the *Cx. pipiens* complex were with, by far, the most abundant species. This correlates with the results of an epidemiological field study conducted in Hamburg, Germany (Krüger et al. 2014), where the *Cx. pipiens* complex (52.28 %, *N* = 10,459) was the most abundant species, followed by *Ochlerotatus annulipes*/*Ochlerotatus cantans* (19.73 %) and *Ochlerotatus communis* (5.45 %). However, in the city of Osijek, Croatia (Merdić et al. 2010), the authors found that *Cx. pipiens* (5.9 %, *N* = 207,136) was amongst the most abundant species in the city, but *Ae. vexans* (75.6 %) and *Oc. sticticus* (13.3 %) were captured at even higher frequencies. The large areas of floodplains surrounding Osijek may explain this discrepancy. The high abundances of the *Cx. pipiens* complex also concur when compared to results from the USA, where this species was found in high abundances in urban areas (Pecoraro et al. 2007; Trawinski and MacKay 2010). *Cx. pipiens* is a potent vector for a variety of diseases. This species complex is assumed to be the main vector for the WNV in Europe (Hubálek 2008). They do not only entail the enzootic amplification of WNV among avian hosts, but also act as bridge vectors to mammalian hosts (Andreadis 2012). In Austria, WNV (lineage 2) was detected for the first time in 2008 in birds of prey (Wodak et al. 2011) and was later isolated from *Cx. pipiens* (Bakonyi et al. 2013). As with *Cx. pipiens*, the main vector species is present in high numbers. It can be proposed that in the future, WNV will spread further resulting in increasing numbers of infections. Another emerging virus in Europe, the USUV, was isolated from *Cx. pipiens* in Austria, and similarly to WNV, it seems that this species is the main vector for this disease in Europe (Weissenböck et al. 2007; Calzolari et al. 2010). From 2001 to 2005, there were outbreaks of USUV during the summer months in Vienna and the surrounding districts, causing mass mortality in birds (Chvala et al. 2007). The outbreak in 2003 was facilitated by an extraordinary hot summer, and it is likely that similar climatic conditions could initiate further USUV outbreaks (Brugger and Rubel 2009). Recently, Sonnleitner et al. (2014) isolated Tahyna virus (TAHV) from *Cx. pipiens* complex pools collected in the Alps (Province of Tyrol, western Austria). The main vertebrate hosts for TAHV are small mammals, but humans can be infected as well, were it causes an influenza-like illness (Hubálek 2008). As reported by Turell (2012), the *Cx. pipiens* complex has also been shown to transmit the Sindbis virus (SINV). Although in Austria SINV has not yet been isolated directly, antibodies against this virus have been found in domestic as well as in wild animals (Sixl et al. 1973). Further, *Cx. pipiens* is an important vector in the transmission of parasitic nematodes causing dirofilariasis (Cancrini et al. 2006; Simón et al. 2012). Possible autochthonous cases of infections with *Dirofilaria repens* have recently been documented in Austria in humans, dogs, and mosquitoes of the genus *Anopheles* (Auer and Susani 2008; Löwenstein and Spallinger 2009; Silbermayr et al. 2014). In Portugal, avian malaria was isolated from *Cx. pipiens* (Ventim et al. 2012). Recently, these pathogens were also confirmed in Austria (Seidel et al. 2013).

*Cq. richiardii*, the second most common species in this study, is known to transmit WNV, SINV, and Batai virus (BATV) and is considered as a possible bridge vector between mammalian and bird hosts (Hubálek 2008; Toma et al. 2008). Although in Austria *An. maculipennis* seems to be the main vector for BATV, it has been isolated from *Cq. richiardii* as well (Aspöck 1968). A high prevalence of 68 % was found in a previous study on cattle in the province of Burgenland (Aspöck and Kunz 1971), but there are no recent data available on the spread of BATV in humans or animals in Austria. *Cq. richiardii* is a vector for TAHV (Aspöck et al. 1970). Results presented by Cancrini et al. (2006) suggest that this species could also transmit dirofilariasis.

*An. plumbeus* breeds mainly in tree holes of deciduous trees (Becker et al. 2010). It has been shown that this species is a vector for malaria (*Plasmodium vivax* and *Plasmodium falciparum*; Solkolova and Snow 2002; Schaffner et al. 2012). In Austria, tertian human malaria was endemic until the beginning of the twentieth century, and small outbreaks occurred until the Second World War. Due to the effective health care system in Austria, a re-emerging human malaria is unlikely, although several anopheline vector species occur in this country (Wernsdorfer 2002; Takken et al. 2007; Lebl et al. 2013). Even when considering increasing temperatures due to climate change, warmer temperatures of a few degrees will not necessarily lead to malaria epidemics (Becker 2008).

*Ae. vexans* are predominately found in flooded areas where they breed in temporary water bodies (Becker et al. 2010). TAHV and USUV have been isolated from *Ae. vexans* in Austria (Aspöck and Kunz 1966; Weissenböck et al. 2007). This mosquito is therefore mainly of concern in the transmission of diseases in wild animals. However, its importance is likely to be higher outside urban areas where their preferred breeding sites are more common and the abundance of wild animals is higher. Outside of Austria, *Ae. vexans* has been shown to serve as a vector for WNV (USA; Hayes et al. 2005) and for dirofilariasis (Slovakia; Bocková et al. 2013).

Similar to *Ae. vexans*, the preferred breeding sites of *Oc. sticticus* are temporary water bodies after floodings (Becker et al. 2010). In Austria, no pathogens have been isolated from this species. However, there are known TAHV vectors in the Czech Republic and Slovakia (Danielová and Holubova 1977) and WNV vectors in the USA (Andreadis et al. 2004).

Comparing the results from this study with those conducted previously in the hinterland of Vienna, a high deviation in the species compositions was observed. Zittra (2013) found in the same floodplain forest we sampled (Danube Floodplain), but at a distance of ∼25 km from Vienna, that *Ochlerotatus geniculatus* (40.3 %, *N* = 221) were most abundant, followed by *Cx. pipiens* (23.1 %), *Cx. richiardii* (19.9 %), and *Culex territans* (10.4 %). Aspöck (1969) also captured mosquitoes in this floodplain forest outside Vienna and found *Oc. sticticus*, *Ae. vexans*, and *Cx. pipiens* to be the dominating species. Those results concur with Šebesta et al. (2010), who captured in another floodplain forest (Soutok area, at a distance of ∼65 km from Vienna) mostly *Ae. vexans* (86.3 %, *N* = 5353), *Oc. sticticus* (6.3 %), and *Cx. pipiens*/*torrentium* (4.5 %). However, the capture rates of species breeding predominantly in temporary water bodies, like *Ae. vexans* and *Oc. sticticus* are likely to vary at a high rate according to the availability of breeding sites before the capture event. Thus, the species composition in floodplain forests is also strongly influenced by the weather conditions prior the capture. Furthermore, Aspöck (1969) collected mosquitoes at the Leithagebirge (~35 km from Vienna), where the most common species were *Oc. annulipes*, *Oc. communis*, and *Ochlerotatus cataphylla*, and near Lake Neusiedl (~50 km from Vienna), where *An. maculipennis*, *Cq. richiardii*, *Ochlerotatus dorsalis*, *Ochlerotatus caspius*, and *Cx. pipiens* were the most abundant species. Those two habitat types are, however, likely to vary profoundly from the habitats in Vienna and are thus difficult to compare with this study. Even so, comparisons of species compositions reveal the importance of mosquito samplings within cities. Using mosquito data from the cities’ hinterland may lead to biased conclusions concerning the risk of mosquito-borne diseases.

## Conclusion

A vector-borne disease can only pose as a threat when the vector species is present in adequate densities. The present study showed that the *Cx. pipiens* complex followed by *Cq. richiardii* are the most common species in the city of Vienna and thus diseases passed on by those mosquito species are likely to be transmitted at higher rates (Table 2). WNV seems to represent the highest threat, for the following reasons: (i) it can be transmitted by several of the most abundant species in Vienna, (ii) it has already been shown to pose a higher risk in cities (Deichmeister and Telang 2011), (iii) it has the possibility to cause severe illness, and (iv) it is already wide-spread in Europe (Bakonyi et al. 2013). Further, this disease has been shown to cause high economic costs and will thus be in the focus of public health authorities (Zohrabian et al. 2004). BATV, SINV, and TAHV are of less concern, as they are not transmitted by *Cx. pipiens* complex and/or cause, at least in humans, less severe illness (Hubálek 2008). However, a more precise assessment of those diseases remains problematic, as published data on cases in Austria date back decades. USUV is likely to re-emerge in Austria only at favorable climatic conditions, i.e., hot summers (Brugger and Rubel 2009). However, a climatic change towards a warmer climate in Europe might facilitate the further spread of this disease, also in Vienna. The increasing number of diagnosed cases of dirofilariasis in humans and dogs indicate that this disease is spreading in Europe (Szénási et al. 2008). Those findings are supported by Sassnau et al. (2014), who showed the principal climatic suitability of certain German regions for the establishment of natural dirofilarial transmission cycles. Several cases have been reported for the city of Budapest, the capital of Hungary (Szénási et al. 2008), indicating that dirofilariasis can occur in urban environments. The distance between Vienna and Budapest is only about 200 km, which makes the occurrence of this parasite in Vienna very likely.

**Table 2:** Confirmed vector-borne diseases in Austria and transmissibility by the most abundant mosquito species captured during this study

	*Cx. pipiens* complex	*Cq. richiardii*	*An. plumbeus*	*Ae. vexans*	*Oc. sticticus*
BATV		x			
SINV	o	o			
TAHV	x	x		x	o
USUV	x			x	
WNV	x	o		o	o
*Plasmodium* sp.	o		o		
*Dirofilaria repens*	o	?		o	

*x* confirmed vector in Austria, *o* confirmed vector somewhere else, *?* suspected vector

For a better estimation of the threat of mosquito-borne diseases in Europe, further studies on the occurrence of mosquitoes in urban environments are necessary. Detailed information on species composition and abundance is essential for epidemiologists, e.g., to model outbreak scenarios to estimate the risk of the (re-)emergence or spread of mosquito-borne diseases. Those risk assessments provide important information for public health authorities, as they have to prioritize the threat of diseases and can use this as a basis to initiate preventing and control strategies.
